# Modified mRNA Therapeutics for Heart Diseases

**DOI:** 10.3390/ijms232415514

**Published:** 2022-12-08

**Authors:** Ajit Magadum

**Affiliations:** Center for Translational Medicine, Temple University, Philadelphia, PA 19140, USA; ajit.magadum@temple.edu

**Keywords:** modRNA, gene therapy, myocardial infarction, heart failure, cardiomyocyte proliferation, apoptosis, inflammation, oxidative stress, cardiovascular disease

## Abstract

Cardiovascular diseases (CVD) remain a substantial global health problem and the leading cause of death worldwide. Although many conventional small-molecule treatments are available to support the cardiac function of the patient with CVD, they are not effective as a cure. Among potential targets for gene therapy are severe cardiac and peripheral ischemia, heart failure, vein graft failure, and some forms of dyslipidemias. In the last three decades, multiple gene therapy tools have been used for heart diseases caused by proteins, plasmids, adenovirus, and adeno-associated viruses (AAV), but these remain as unmet clinical needs. These gene therapy methods are ineffective due to poor and uncontrolled gene expression, low stability, immunogenicity, and transfection efficiency. The synthetic modified mRNA (modRNA) presents a novel gene therapy approach which provides a transient, stable, safe, non-immunogenic, controlled mRNA delivery to the heart tissue without any risk of genomic integration, and achieves a therapeutic effect in different organs, including the heart. The mRNA translation starts in minutes, and remains stable for 8–10 days (pulse-like kinetics). The pulse-like expression of modRNA in the heart induces cardiac repair, cardiomyocyte proliferation and survival, and inhibits cardiomyocyte apoptosis post-myocardial infarction (MI). Cell-specific (cardiomyocyte) modRNA translation developments established cell-specific modRNA therapeutics for heart diseases. With these laudable characteristics, combined with its expression kinetics in the heart, modRNA has become an attractive therapeutic for the treatment of CVD. This review discusses new developments in modRNA therapy for heart diseases.

## 1. Introduction

Cardiovascular diseases (CVD) are significant causes of death worldwide, and more people die annually from CVDs than from any other disease. A measured 17.9 million people died from CVDs in 2016, which was 31% of global mortality [1]. It is expected that there will be over 23 million deaths from CVDs annually, by 2030. About 5.8 million people in the United States suffer from heart failure (HF), and nearly 700,000 new cases are diagnosed yearly. The global cost of CVDs will reach above USD 1 trillion by 2030 and is a tremendous pressure on the economy [2]. There have been no drugs or therapeutics approved by the FDA in the last 20 years to treat the underlying cause of the disease (ischemic heart diseases and HF). The medication for HF most likely includes off-patented angiotensin-converting enzyme (ACE) inhibitors, vasodilators, beta-blockers, diuretics or water pills, and digoxin. All these medications help maintain heart function for a short time, but do not cure it. HF is a complex disease divided into three subtypes based on the left ventricular ejection fraction (LVEF): HF with reduced ejection fraction (HFrEF), of less than 40%; HF with mid-range ejection fraction (HFmrEF), 40% to 49%; and HF with preserved ejection fraction (HFpEF), 50% or higher. HFrEF usually originates because of an ischemic condition, while hypertension and atrial fibrillation are associated with HFpEF. β-blockers and ACE inhibitors help lower mortality in patients with HFpEF and HFmrEF, while other vasodilators, diuretics, or digoxin augment heart function but do not reduce the mortality rate [3]. These approaches for MI or HF do not treat the primary cause of the disease. An estimated 20–40% of cardiomyocytes (CMs) in the heart die post-MI, which drives the heart to a constantly reduced ejection fraction and leads to HF. Failing to efficiently replace the lost CMs in the heart after MI and HF is a principal task to address, to improve clinical outcomes and survival [1,4]. It was believed for a long time that the adult mammalian heart does not regenerate, but recent studies in mice have shown that the mammalian heart can regenerate just after birth. However, it loses its capacity to regenerate in the first week (7 days) post-birth [5]. Multiple gene expression studies have been conducted in a narrow window of heart regeneration to find the genes significantly differentially expressed between these time points [5,6]; the mitosis and cell cycle genes were found to be mostly differentially expressed. In the last two decades, several studies have shown that mammalian CMs can be induced to proliferate by proliferative genes in vitro and in animals by using proteins, viruses, small molecules, or transgenic mouse models [7,8,9,10,11,12,13,14]. However, these methods have limitations, including very short or long half-life, complicated local administration, lack of CMs specificity, the inability to deliver intracellular genes such as transcription factors (proteins and small molecules), and a very long and sustained uncontrolled expression that may lead to increased, unrestrained CMs size and cardiac hypertrophy, edema, and arrhythmia (viruses) [15,16,17]. In contrast, transgenic mouse models are just for studies and cannot be used for therapies. Therefore, a substantial unmet need exists to develop novel, clinically appropriate therapeutics for heart diseases to repair the injured cardiac tissue and reverse the pathological remodeling.

In the last three to four decades, hundreds of gene therapy clinical trials have been conducted worldwide. Only seven gene therapy programs were approved by the European Medicines Agency or Food and Drug Administration (FDA) until 2019. A few hundred are in the last phases of the clinical approval pipeline. The first gene-drug approved was Glybera, for treating the genetic disorder severe lipoprotein lipase deficiency. This provides hope for the development of gene therapies for different CVDs. Despite great enthusiasm and positive preclinical results, clinical translation of cardiovascular gene therapy has yet to be very successful due to low transfection efficiency, short half-life or expression, long-term expression of the transgene, development of CH and arrhythmias, inadequate knowledge about underlying pathophysiological mechanisms and genes, and the poor design of clinical trials. The peculiar characteristics and uses of different gene delivery approaches for the heart are listed in Table 1. Recently, RNAs have developed as a promising therapeutic possibility for many human diseases, including CVD [18,19,20]. The function of non-coding RNAs, including long non-coding RNAs (lncRNAs), microRNAs (miRNAs), and circular RNAs (circRNAs), has only begun to arise in the past 10–15 years [19]. The synthetically synthesized siRNA (small interfering RNA), shRNA (short-hairpin RNA), antisense oligonucleotides (ASOs), short activating RNA (saRNA), RNA aptamers, to single guide RNA (sgRNA) for CRISPR/Cas9 systems, were used for quite a time in clinical studies. In 1998, the first RNA drug (antisense RNA) was approved, called fomivirsen, for CMV retinitis. Furthermore, in 2016, nusinersen, a splicing switching ASO, was approved by the FDA and became the first drug to treat spinal muscular atrophy. However, the recent approval of two siRNA-based drugs by the FDA, patisiran and, givosiran, and mRNA-based vaccines against COVID-19, gained momentum in clinically developed RNA therapeutics [21,22]. Given advantages such as ease of dosage control, low immunogenicity, and no danger of genome integration, nucleic acid-based products have gained significant attention in recent times as a potential therapeutic approach. A PCSK9-targeted siRNA, called Inclisiran, is in its phase III trial to reduce LDL, has a clean safety signature for the heart, and was recently approved by the European Commission. Similarly, an antisense oligonucleotide targeting an LPA mRNA, called AKCEA-APO(a)-L, reduced the lipoprotein(a) concentrations below 50 mg/dl (safe level) in more than 90% of patients in its phase II trial [23]. 

**Figure 1:**
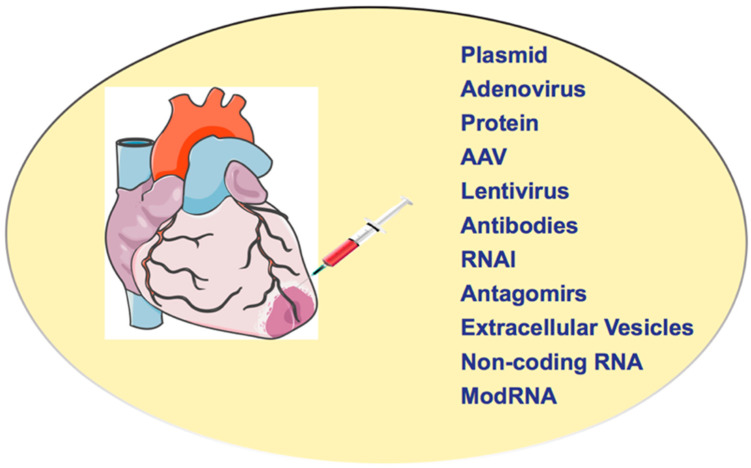
**Methods to deliver the genetic material in the heart in vivo.** Adenovirus, AAV, lentivirus, recombinant proteins, and antibodies were previously developed to target the heart in animal models and clinical settings. In contrast, RNA-based methods were developed recently, including RNA interference (RNAi and shRNA) (a process where RNA molecules inhibit gene expression or translation by neutralizing targeted mRNA molecules), microRNA (endogenous, a small non-coding RNA molecule (20-22 nucleotides) post-transcriptionally regulate gene expression), non-coding RNAs (ncRNAs) (RNA molecules which do not translate into a protein), antagomirs (chemically synthesized oligonucleotides that inhibit miRNA binding to the desired site on an mRNA molecule), and modRNA.

mRNA is a single-stranded RNA molecule that matches the genetic sequence of a gene and is read by a ribosome to synthesize a protein. The mature mRNA is formed after the transcription of DNA and processing of pre-mRNA [24]. Even though mRNA was discovered in 1961, the first successful demonstration of mRNA delivery to cells using liposomes was provided by Malone et al. in 1989 [25,26]. Wolff et al., in 1990, showed that direct mRNA delivery and its translation into protein could be possible in mouse skeletal muscle [27]. However, mRNA-based studies remained limited due to their instability (mRNA is prone to cleavage by RNase and can trigger the innate immune system) until the pioneering work by Karikó et al. in 2008 [28]. Substitution of uridine residues in mRNA with the readily occurring modified nucleoside pseudouridine (the uracil is attached via a carbon–carbon instead of a nitrogen–carbon glycosidic bond) magnified translation due to changes in the secondary structure of the mRNA, restricting its recognition by the TLRs and nucleases [28,29]. The modRNA is a prompt (translated within minutes), highly effective (high expression), dose controlled (expression controlled by the amount of modRNA used), transient (pulse-like kinetics for 6–8 days), safe, higher RNase resistance, no genomic integration, efficient translation, and without innate immunity. modRNA is synthesized with 100% replacement of uridine by N1-Methylpseudouridine-5’-Triphosphate (1- mψU) [12,13,15,28,29,30,31,32,33,34]. In cardiac cells and the heart, modRNA translation starts in minutes, peaks between 12 and 48 h, and declines by 48–120 h (transient expression). As modRNA is a nucleotide sequence, its expression level in the cell can be controlled very well by the concentration of modRNA used for transfection. As modRNA cannot enter the nucleus and integrate into the genome, it does not create genomic mutations and, consequently, does not affect the function [28,35]. The delivery of external genetic material, including mRNA, into the cell, as the cell recognizes it as external, and activates TLR receptors and RIG proteins to induce innate immunity [28,29], while modifications such as m1Ψ and 5 mC change the secondary structure of modRNA so that the TLR receptors cannot recognize it and RNases cannot degrade it.. The modifications m1Ψ and 5 mC cause modRNA to be more resistant to RNases and to inhibit innate immune response, thus, modRNA is translated very efficiently and subsequently stabilized. The different gene delivery methods to the heart or cardiac muscle are shown in Figure 1 [12,13,15,32,33,34,35,36,37]. The efficiency of modRNA delivery in vivo has been increased by enhancing the stability of mRNA and increasing translational efficiency by capping the molecule with 30-O-Me-m7G (50) ppp (50) G Anti Reverse Cap Analog (ARCA) at its 5^1^ ends and, most recently, cleanCap [38]. The uses (published studies) of modRNA technology as a gene therapy tool for heart diseases are listed in Table 2.

## 2. modRNA Technology Development and Delivery

The in vitro synthesis of modRNA usually involves four steps: (1) creation of the DNA template with the desired transcript; (2) in vitro transcription (IVT) of the desired DNA template; (3) 5′ phosphates of modRNA removal with Antarctic phosphatase treatment; and, (4) precipitation of modRNA using 5 M ammonium acetate salt. Current modRNA synthesis protocols such as cleanCap involve three main steps: (1) creation of the DNA template with the desired transcript; (2) in vitro transcription (IVT) of the desired DNA template; and, (3) purification of modRNA. To concentrate the modRNA for in vivo use, ammonium acetate precipitation or ultracentrifugal filters are applied to the samples. 

To develop effective gene therapy for the heart, the delivery systems carrying the nucleic acid must ensure the following:

1. The optimum uptake of the nucleic acid by cardiac cells;
2. The optimum expression and time of expression of nucleic acid;
3. Minimum or no immune response in the heart;
4. Efficient translation, stability, and transfection of the gene in the heart;
5. The delivery material should be safe and not interfere with gene function.

Selecting a suitable delivery method to deliver modRNA successfully into the heart is critical as it cannot directly diffuse into the negatively charged cardiac cells. The modRNA can be transfected well in vitro by complexing it with positively charged transfection reagents such as lipids, nanomaterials, or polymers. This complex allows it to attach to the cell membrane and consequent endocytosis. RNAiMAX, a lipid-based transfection reagent, is widely used to transfect cells with RNA. RNAiMAX induces stable and significant transfection of human-induced pluripotent stem cell (hiPSC)-derived CMs in vitro. TransIT (<90% transfection), JetMESSENGER (<90% transfection), and MessengerMax (<80% transfection) also induce stable modRNA (m1Ψ) transfection in cardiac cells [33]. For in vivo transfection of mouse hearts, Zangi et al. showed that the vascular endothelial growth factor a (VEGFa)/Beta-galactosidase (B-gal)/Luciferase (Luc) modRNA delivery to the heart using RNAiMAX transfection reagent induced significant modRNA (m1Ψ) translation for a week. This allowed study of the effect of VEGFa on the heart post-MI [35]. Huang et al. also used RNAiMAX to deliver IGF1 into the mouse heart post-MI, showing it induced cardiac protection. In contrast, using RNAiMAX in the heart was associated with an increased level of apoptotic cells around the injection site in the myocardium [34]. Thus, choosing an appropriate delivery method is critical for the transfection of cardiac cells in the heart in vivo. Turnbull et al. used formulated lipidoid nanoparticles (FLNPs) and evaluated the modRNA (m1Ψ + m5C) transfer [15,44]. They demonstrated that FLNPs-delivered mRNA (GFP) translated into protein in 20 min in the rat and pig myocardium. Sultana et al., by using different available transfection reagents in the market, showed that naked modRNA in sucrose–citrate buffer translated modRNA (Luc or GFP modRNA) very efficiently, fast (detected within 10 min in cardiac muscle) and had stable protein expression for days [33]. The injection modRNA using this buffer achieved more than 20% of the left ventricle transfection (LV). The modRNA delivery platform will allow the expression of functional genes to cure heart diseases. Use of RNAiMax and other lipid-based transfection reagents showed an increase in cell apoptosis compared with the sucrose–citrate buffer, suggesting the buffer itself is not harmful to the heart. Similarly, Carlsson et al. used the saline–citrate buffer to deliver VEGFa modRNA in the swine heart and found significant expression of VEGFa protein, which, in turn, significantly improved cardiac function post-MI [40]. Recently Singh et al. showed that the delivery of M3RNA (microencapsulated modified messenger RNA), cardiac fibroblasts, and primary CMs, induced protein expression as early as 2–4 h and lasted up to 7 days [42]. Myocardial delivery of firefly luciferase (FLuc) and mCherry M3RNA in mice using nanoparticles resulted in FLuc or mCherry protein within 2 h, sustaining for 72 h. In a porcine model of MI, intracoronary delivery of alginate carrying M^3^RNA encoding mCherry resulted in rapid protein expression in porcine hearts. 

Many researchers worldwide are trying to increase the translational capacity and stability of modRNA. Enhancing translation efficiency in the ischemic heart is valuable as it can reduce the modRNA needed per delivery and achieve higher and longer protein production post-single delivery. To achieve this, a few properties of mRNA or modRNA can be modulated or changed for better expression, for example, 51 untranslated regions, 31 untranslated regions, Poly A tail, better codon usage, and chemical modification of nucleotides. Recently, we studied the dynamics of heart LV transcriptome and proteome post-MI and analyzed the changes in gene expression and protein levels in the LV of mice 4 and 24 h post-MI and compared them to LV from sham-operated mice [37]. Several genes were identified that were differentially expressed and had a different 5′UTR sequence. Next, we screened and identified that the 5′UTR of a gene called carboxylesterase 1D (Ces1d), which is known to be involved in fatty acid metabolism, enhanced the translation of Luc modRNA (m1Ψ) two-fold in the heart post-MI. Adding 5′UTR of Ces1d in the reporter mRNA (Luc or GFP) induced higher and longer protein expression. Element D, an RNA element in the Ces1d gene, improved modRNA translation and increased, by 2.5-fold, translation over Luc modRNA carrying synthetic 5′UTR post-MI [37]. Hadas et al. developed an in vitro modRNA synthesis protocol, which presented a higher modRNA yield [41]. They showed that changing the ratio between the anti-reverse cap analog (ARCA) and N1-methyl-pseudouridine (N1mΨ), favoring ARCA over N1mΨ, significantly increased the yield per reaction, improved modRNA translation, and reduced its immunogenicity in vitro [41]. This protocol will make modRNA preparation more accessible and financially affordable for basic and translational research.

## 3. Cardiomyocyte-Specific modRNA Expression in the Heart

**Figure 2:**
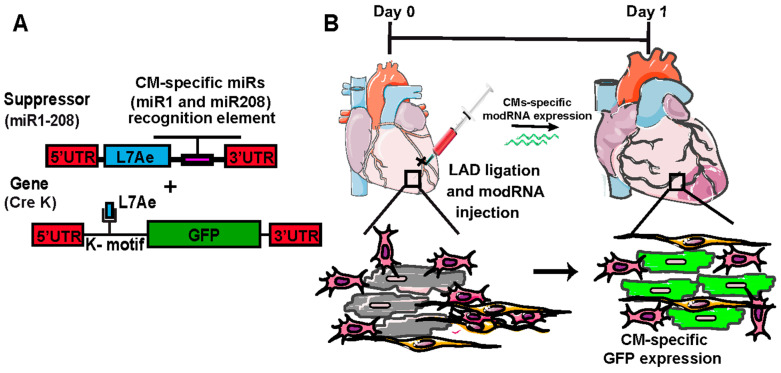
**CM- and non-CM-specific modRNA expression platform.** (**A**) Experimental design using two modRNA-based constructs to deliver any gene exclusively in CMs. (**B**) Schematic diagram showing CM-specific GFP modRNA expression in the mouse heart 1 day after MI and myocardial delivery of both modRNA constructs (Green wavy line represents modRNA which codes for gene of interest (GFP) would be delivered).

The use of cell cycle inducers such as cyclins, viral proteins, and growth factors in a non-cell-specific (global) manner increases proliferation in CMs and non-CMs, which might inhibit the cardiac repair process. To express any gene (modRNA) only in CMs, a CM-specific modRNA system has been designed that has two distinct modRNA constructs [13]. The first construct is a suppressor modRNA (m1Ψ), which carries L7AE, an archaeal ribosomal protein that regulates the translation of a modRNA (gene) of interest with a kink-turn motif (k motif), a specific binding site for L7AE [46,47]. Translation of L7AE modRNA suppresses the translation of the modRNA (gene) of interest when the two are co-transfected into the cell. By adding a CMs-specific microRNA (miR) recognition element to the L7AE gene, we can prevent L7AE translation in CMs that plentifully, and mostly exclusively, express the miR (“suppress the suppressor” approach), allowing the precise translation of the modRNA (gene) of interest in CMs. To develop the platform, it is essential to find the miRs specific to CMs. miR-1, miR-208, and miR-199 are expressed mostly in CMs [48,49,50]. We used modRNA-based reporter constructs to find that both miR-1 and miR-208 were CM-specific miRNAs [13]. To develop the CM-specific modRNA expression platform, we designed an L7AE modRNA that carried both miR-1 and miR-208 recognition elements (L7AE miR-1 + miR-208) and generated a nuclear GFP modRNA (m1Ψ) (nGFP—k-motif) that included the k-motif (L7AE recognition site) (Figure 2A). Using our adult mouse MI model, we showed that the transfection of the only nGFP—k motif resulted in the translation of nGFP in both CMs and non-CMs. However, when nGFP—k motif was co-transfected with L7AE miR-1 + miR-208, only CMs expressed the nGFP protein, suggesting that the system worked (Figure 2B). Intra-myocardial delivery of Cre K modRNA (m1Ψ) (a destabilized Cre recombinase (DD-Cre—k motif) modRNA) using Rosa26 reporter mice (Rosa26mTmG) resulted in GFP expression (switch) in CMs and non-CMs covering ~50% of the LV post-MI; however, when co-transfecting Cre-K with miR-1-208 (_CMS_Cre), GFP^+^ CMs covered ~20% of the LV post-MI [13]. Additionally, there was no GFP expression in other organs (spleen, lung, or liver), supporting the cell specificity of the platform. The CM-specific Pkm2 modRNA (m1Ψ) expression significantly induced the CM cell cycle 7 days post-MI without affecting the non-CMs. In contrast, the global expression of Pkm2 induced both CM and non-CM cell cycles compared with the control (Luc). As discussed above, the CM-specific delivery of Pkm2 modRNA significantly improved cardiac function, CM proliferation, mouse survival, and reduced scar size compared with the global Pkm2 modRNA post-MI [13]. As miR-1 and miR-208 sequences are very conserved between species, the CMs-specific modRNA tool can be applied for large animal studies and in the clinic.

## 4. modRNA in Cardiomyocyte Proliferation

Cell proliferation is the process where cells undergo cell division that increases the number of cells. The balance between cell divisions and cell loss through cell death or differentiation creates a homeostatic state in the organ. Hearts lose approximately 20–40% of CMs after MI, which puts enormous pressure on the heart to pump blood effectively. Lower vertebrates such as zebrafish and newts induce cardiac regeneration after injury by inducing the proliferation of resident CMs [51,52]. Recently, Porrello et al. showed that 1-day-old mice could regenerate their heart after injury, but not 7-day-old mice [5]. Using cell lineage experiments, the researchers showed that CM proliferation induced new muscle formation. In the last two decades, numerous efforts have been undertaken to overexpress positive regulator cell cycle proteins, growth factors, genes, viral proteins, and non-coding RNAs, and inhibit negative regulators of the cell cycle from inducing CM proliferation and cardiac regeneration, but this has resulted in limited success in animal studies. Novel genes and molecular pathways have shown the robust induction of CM proliferation in mouse models of heart diseases [7,8,13,14,53,54,55,56]. The partial success of bringing them into a large animal study and clinic is due to the limited proliferation potential of a gene, genes, or molecular pathway, and inadequate platform of gene delivery methods to the heart. 

**Figure 3:**
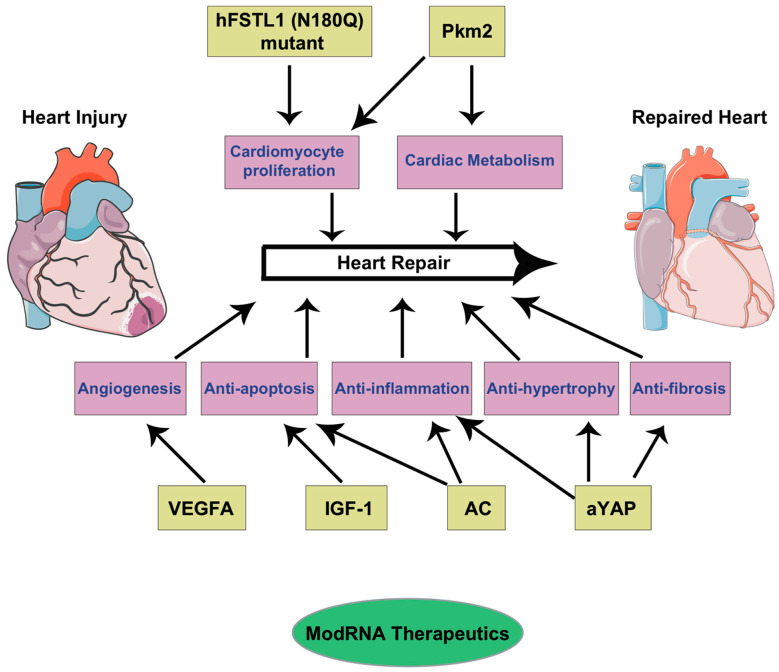
**The delivery of modRNA based genes to heart to induce heart repair.** Genes such as Pkm2, VEGFa, AC, aYAP, IGF1, and hFSTL1 (N180Q) mutated in the form of modRNA induce heart repair through cardiomyocyte proliferation, angiogenesis, cardiac protection, modulation of inflammation and cardiac metabolism, and inhibition of apoptosis and cardiac fibrosis.

Pkm2, also known as pyruvate kinase muscle isozyme 2, is a glycolytic enzyme [57]. Pyruvate kinase dephosphorylates phosphoenolpyruvate to pyruvate and produces net ATP. Pkm2 is highly expressed in proliferating cells such as embryonic and tumor cells, and in differentiated tissues such as lung, fat tissue, retina, and pancreatic islets [58]. Recently, we showed that Pkm2 expression was high in the mouse heart during embryonic development, reduced significantly with development (lower expression in the P8 heart), and was relatively less expressed in the adult heart [31]. Pkm2 expression correlates with a decrease in CM proliferation with heart development. The overexpression of Pkm2 modRNA (m1Ψ) in P3 or P4 rat neonatal CMs induced CM proliferation, analyzed by the immunostaining of cell cycle markers such as BrdU incorporation, ki67, pH3, and aurora B, and cell division by time-lapse imaging. The peculiar characteristics of CM cell division, such as sarcomere disassembly, were also observed after Pkm2 modRNA expression, where sarcomere myofibrils were disassembled to allow cells to undergo mitosis and cytokinesis. Pkm2 expression in cardiac cells was transient, started expressing a few hours after transfection, and stayed for more than a week [31]. Next, we studied the expression pattern of Pkm2 and its effect on CM proliferation and cardiac regeneration. The delivery of different modRNA-induced heart repairs is shown in Figure 3.

The myocardial injection of Pkm2 modRNA induced transient and stable protein expression for 8–12 days. The expression of Pkm2 modRNA induced cell cycle activity in both CMs and non-CMs post-MI [31]. It is well known that induction of fibroblast or immune cell proliferation post-MI inhibits the regeneration process and cardiac function improvement. Therefore, we developed CM-specific SMRTs to eliminate the disadvantages of pro-proliferative gene expression in fibroblast and immune cells, which are known to inhibit cardiac regeneration post-myocardial injury. Myocardial delivery of _CMS_Pkm2 modRNA-induced CM cell cycle (pH3^+^ or BrdU^+^ or ki67^+^ CMs) was achieved without affecting the non-CMs 7-days post-MI. The global expression of Pkm2 modRNA in the heart significantly induced the CM cell cycle, compared with the control Luc modRNA, but to a lesser extent compared with _CMS_Pkm2 modRNA, while the global expression of Pkm2 significantly induced the non-CM cell cycle [13]. Twenty-eight days post-MI and injection of _CMS_Pkm2 modRNA in the myocardium improved the cardiac function, suggesting that _CMS_Pkm2 delivery induces long-term positive outcomes compared with the control, modRNA Luc [13]. There were increases in capillary density in _CMS_Pkm2 modRNA-injected mouse hearts. The scar size was reduced significantly in _CMS_Pkm2 modRNA-injected mouse hearts, which was also supported by the increased heart-to-body weight ratio and considerably smaller CM size. This suggests that _CMS_Pkm2 modRNA does not develop hypertrophy but induces CM hyperplasia and reduces the scar size post-MI. As a result of improved cardiac function, CM proliferation, and induced angiogenesis, the survival rate of _CMS_Pkm2 modRNA-injected mice was significantly higher than Luc-injected mice post-MI. We also looked at the effect of Pkm2 modRNA in the HF model, where _CMS_Pkm2 or _CMS_Luc modRNA was injected in the myocardium for 15 days post-MI. The cardiac function was significantly increased in _CMS_Pkm2 modRNA-injected mice 1 month post modRNA injection. This was supported by the increased CM cell cycle and heart-to-body weight ratio in these mice, suggesting that _CMS_Pkm2 modRNA improved cardiac function in the HF model. Overall, _CMS_Pkm2 modRNA induced CM proliferation, reduced scar size, improved cardiac function, and mouse survival post-MI and HF. 

Recently, we showed the role of hFSTL1 (human follistatin-related protein 1) glycosylation site mutation in CM proliferation, and revealed that the myocardial delivery of a mutated hFSTL1 modRNA (m1Ψ) with a single asparagine-to-arginine (N-Q) substitution in the glycosylation site (N180Q) was essential and necessary to increase the proliferation of neonatal rat CMs in vitro or adult CMs post-MI [12]. FSTL1 has a vital role in cardiac development and also in disease [9]. FSTL1 protein has cardioprotective effects and has been shown to avert myocardial ischemia/reperfusion injury in a mouse or pig model of ischemia/reperfusion. Recombinant FSTL1 protein applied as a patch on epicardium post-MI, induced CM proliferation and cardiac function. It attenuated the hypertrophy mice pressure overload model [59]. We showed that hFSTL1 (N180Q) modRNA did not induce CM hypertrophy post-MI [12]. Twenty-eight days post-MI and myocardial injection of hFSTL1 (N180Q) induced significant improvement of cardiac function (EF, %FS) compared with hFSTL1 or Luc modRNA [12]. The scar size was reduced, and mouse survival increased significantly in hFSTL1 (N180Q) injected mice. This showed that a single injection of hFSTL1 (N180Q) modRNA induced CM proliferation and cardiac regeneration post-MI. These encouraging results of using modRNA platforms to induce CM proliferation will help to recapitulate neonatal mouse cardiac regeneration, and can help to develop therapeutics for CVD in the clinic.

## 5. Cardiomyocyte Apoptosis and Survival

Proliferation, cell survival, and death are vital for organ growth, homeostasis, and pathogenesis. After MI, specifically, CMs undergo stress without oxygen and nutrients, resulting in their apoptosis and necrosis. Millions of CMs die in the next few days post-MI in mammals, which results in pressure on the remaining heart or CMs to effectively pump the blood. Apoptosis starts from two major signaling pathways, intrinsic and extrinsic [60]. In the last two to three decades, much advancement has occurred toward elucidating the apoptotic molecular signaling pathways or genes in CMs, for example, the phosphatidylinositol 3-kinase (PI3K)/protein kinase B (Akt) pathway (pTEN, GSK-3, pim1, Fox01, and PHLPP), mitogen-activated protein kinases (MAPK) pathway (ERK1/2, SAPKs), Integrin/FAK, tumor necrosis factor-α (TNF-a)/NF-kB, GPCR, Hippo pathway, small GTPases, PKC, PKA, cell cycle regulators, jagged/notch signaling, and Calcineurin CaMKII, etc. [60]. Recently, Hadas et al. showed that acid ceramidase (AC) has anti-apoptotic effects post-MI [36]. Ceramides are simple membrane sphingolipids, and high cellular ceramide levels can trigger programmed cell death [61]. The elevated ceramide concentrations in plasma are correlated with a higher possibility of MI reoccurrence and death. Ceramide levels are high in the heart tissues of rodents and humans during acute MI [62,63,64]. Blocking ceramide synthesis in mice and rats can improve cardiac function post-MI [63,64]. Ceramidases such as AC hydrolyze ceramide to generate free fatty acids and sphingosine, which is then phosphorylated by sphingosine kinase (Sphk) to produce sphingosine 1-phosphate (S1P), a pro-survival lipid mediator with functions in different molecular pathways [65]. CM cell death and cardiac inflammation are vital processes that induce severe cardiac dysfunction post-MI. Hadas et al. showed that AC modRNA (m1Ψ) could effectively express AC protein both in vitro and in mouse hearts [36]. In vitro, AC modRNA was delivered into rat cardiac cells and the effect of AC on cell apoptosis under hypoxic conditions was analyzed. The terminal deoxynucleotidyl transferase dUTP nick end labeling (TUNEL) method was used to analyze the apoptosis of cells, which detects extensive DNA degradation as a sign of cell death. The overexpression of AC modRNA inhibited the hypoxia-induced cell apoptosis and increased cell survival compared with the control, Luc modRNA [36]. Next, Hadas et al. analyzed cell apoptosis in a mouse model of MI. The myocardial injection of AC modRNA in mouse heart post-MI significantly (around 50%) reduced the apoptosis (TUNEL positive cells) of CMs and non-CMs in 48 h, which correlated with a reduction in ceramide levels, inhibition of caspase-3 dimer formation and cleavage. These data indeed suggest that AC inhibits cell apoptosis in the heart. As a result, AC modRNA-injected mouse hearts showed an increase in cardiac function (ejection fraction and fractional shortening) 28 days post-MI compared with the control, Luc modRNA [36]. The scar size was significantly reduced; as a result, survival increased dramatically in AC modRNA-injected mice. Overall, the author showed that AC modRNA induced cell survival and cardiac function and improved the survival of mice by inhibiting cardiac cell death after MI in mice. 

The overexpression of CM-specific Pkm2 modRNA in myocardium post-MI reduced CM apoptosis by more than 40%, suggesting its role in cellular apoptotic and survival pathways [13]. Pkm2 induces this anti-apoptotic pathway by inducing the expression of VEGF and reducing oxidative stress. Pkm2 modRNA delivery to the heart or cardiac cells alters the glucose flux from glycolysis towards the PPP. By using a ^13^C glucose metabolic study, it was shown that there was an increase in the number of pentose phosphate pathway (PPP) metabolites, suggesting the activation of PPP. The upregulation of PPP reduces oxidative stress, ROS production, and oxidative DNA damage. The primary sources of ROS in the ischemic–reperfused myocardium are mitochondria, xanthine oxidase, and nicotinamide adenine dinucleotide phosphate (NADPH) oxidase. Increased ROS induces different apoptotic pathways. In prostate cancer cells, the knockdown of Pkm2 induced autophagic cell death via the AKT/mammalian target of the rapamycin (mTOR) pathway [66]. Pkm2 modRNA injection in mouse heart post-MI induced GSH/GSSG levels, while NADP/NAPDH levels reduced, suggesting reduced oxidative stress [13]. Taken together, Pkm2 modRNA injection in the heart post-MI reduced oxidative stress and significantly decreased apoptotic CMs.

Huang et al. showed that the myocardial delivery of insulin growth factor 1 (IGF-1) modRNA (m1Ψ) post-MI induced CM survival and reduced CM apoptosis [34]. Similar results were obtained when IGF-1 modRNA transfected in cardiac cells under hypoxic conditions. The transfection of IGF-1 modRNA with a polyethyleneimine-based nanoparticle complex resulted in protein expression within 2 h and was stable for 48 h. The delivery of this complex decreased TUNEL-positive CMs by more than 50% post-MI. Next, Huang et al. showed that the decrease in apoptotic CMs was due to the activation of the AKT-ERK pathway and high levels of Akt and Erk phosphorylation [34]. The reduction in IGF-1-specific miRNAs was also observed in IGF-1 modRNA-injected mice.

## 6. modRNA in Cardiac Inflammation

After MI, there is extensive apoptosis and necrosis of ischemic CMs, which activates the innate immune response triggering a robust inflammatory response. The dying cells release different types of signals, molecules, and Toll-like receptor (TLR)/interleukin (IL)-1 signaling is stimulated after the injured matrix and activates the complement cascade [67]. This results in the induction of chemokines, cytokines, adhesion molecules, and activation of the nuclear factor (NF)-κB system. Subsequent neutrophils and mononuclear cells are infiltrated in the infarct area of the heart to clear it from dead cells and matrix debris. In the second phase, they activate molecular pathways of cardiac repair [67]. Post-MI actively charged immune response induces cardiac remodeling associated with chamber dilation, systolic dysfunction, and HF. Lately, Hadas et al. showed that myocardial delivery of AC modRNA post-MI modulated the early immune response in the heart [36]. The fluorescence-activated cell sorting (FACS) analysis of the immune cell population from the infarct area 2 days post-MI and AC modRNA-injected mice showed a more than 30% decrease in neutrophils, while the RNAseq analysis of the AC modRNA-injected mouse heart post-MI revealed differential expression of the genes involved in inflammation and neutrophil degranulation. The reduced number of neutrophils in the heart post-MI has been shown to induce cardiac function and reduce scar size [68]. The molecular mechanism by which AC modRNA reduces the neutrophils in the heart was not well illustrated, but the author concluded that it might be through altering the chemokine expression. 

It is well known that the transcriptional co-activator YAP induces CM proliferation and improves survival and myocardial outcome after MI [69]. However, its role in inflammation and cardiac hypertrophy was not previously studied. The author developed a mutated YAP where YAP modRNA (m1Ψ) encoded FLAG-tagged human YAP containing a serine 127 to alanine mutation, which activated YAP (aYAP) by making it resistant to Hippo kinase phosphorylation. In comparing Veh+IR and aYAP+IR hearts, aYAP+IR had much fewer neutrophils (Ly6G+ myocardial area fraction) (12.53% ± 5.67%, versus 29.81% ± 11.02%) and similar cell density [39]. In FACS, CD45+ cells and neutrophils were comparatively less in YAP+IR compared with the control, and also had much fewer neutrophils, suggesting that aYAP primarily reduced the size of the affected region and infiltration of neutrophils two days post-IR [39]. TLR signaling activates the expression of cytokines/chemokines and develops an immune response against injury or infection. The treatment of NRVMs with both LPS or H2O2 and aYAP adenovirus increased the cell viability compared with the control. The LPS-induced cytokines/chemokines (Ccl2, Il-1b, Il-6, Il-10, Il-12a, Il-12b, Il-18, and TNFa) were also down-regulated, suggesting that activation of YAP blocks LPS- or H2O2-induced necrosis and immune response [39]. The author showed that aYAP modRNA improved CM survival through TLR-dependent and TLR-independent pathways, and reduced cardiac inflammation and hypertrophic remodeling after ischemia–reperfusion (IR) stress.

## 7. modRNA in Cardiac Oxidative Stress

In acute MI, reactive oxygen species (ROS) are generated in the ischemic myocardium, especially after reperfusion. ROS directly injure the cell membrane and causes cell death [70]. However, ROS also stimulate signal transduction to elaborate inflammatory cytokines, e.g., TNF-α, interleukin (IL)-1β, and -6, in the ischemic region and surrounding myocardium as a host reaction [71]. Inflammatory cytokines govern cell survival and cell death at multiple levels with ROS. ROS and inflammatory cytokines are cardio-depressants mainly due to impaired intracellular Ca2^+^ homeostasis [71]. Inflammatory cytokines stimulate apoptosis through a TNF-α receptor/caspase pathway, whereas Ca2^+^ overload induced by extensive ROS generation causes necrosis through enhanced permeability of the mitochondrial membrane (mitochondrial permeability transition). ROS stimulate the production of inflammatory cytokines, and inversely, inflammatory cytokines stimulate ROS formation. ROS and inflammatory cytokines activate the matrix metalloproteinases (MMPs) and collagen deposits in the chronic stage, affecting the injured myocardium’s tissue repair and remodeling process. 

The delivery of _CM_Pkm2 modRNA into the heart induces significant expression of glucose-6-phosphate dehydrogenase (G6PD), a rate-limiting glycolytic enzyme of the pentose phosphate pathway (PPP), in comparison with adult CMs transfected with _CM_Luc modRNA [13]. The ^13^C isotopic tracers for metabolic flux analysis showed that Pkm2 modRNA delivery to CMs diverted the glucose flux from glycolysis toward the PPP. There was an increase in the PPP pathway metabolites and nucleotides, reducing oxidative stress, ROS production, and oxidative DNA damage. ROS directly injure the tissue and are critical for inducing cell death. Pkm2 modRNA injection in mice hearts post-MI induced GSH/GSSG (glutathione/glutathione disulfide) levels, and the levels of NADP/NADPH were reduced, suggesting reduced oxidative stress [13]. HPLC analysis showed a significant reduction in superoxide and other ROS levels after CMPkm2 injection post-MI, resulting in reduced oxidative stress and better cardiac function. The substantial reduction in phosphorylated ATM serine/threonine kinase (pATM) and 8- hydroxyguanosine (8-OHG) positive CMs post-Pkm2 modRNA injection after MI suggests a decrease in DNA damage. Taken together, reducing oxidative stress and DNA damage helps cells survive better, and available ribonucleotides provide building blocks for cell proliferation post-Pkm2 modRNA expression in the heart [13].

## 8. modRNA in Cardiac Metabolism

More than 95% of ATP generated in the adult mammalian heart is derived from oxidative phosphorylation in the mitochondria, and the remainder comes mostly from glycolysis [72]. Approximately 70% to 90% of cardiac ATP is produced by the oxidation of fatty acids (FAs). The remaining 10% to 30% comes from the oxidation of glucose and lactate, as well as small amounts of ketone bodies and certain amino acids [72]. During heart development, in the embryonic stage, cardiac metabolism is based mainly on lactate and glycolysis, while after birth, the metabolism shifts more toward oxidative phosphorylation. After MI in adult mice, there is a shift in cardiac metabolism from oxidative phosphorylation to glycolysis with an increased expression of glycolytic genes and a reduction in the expression of oxidative phosphorylation genes.

Pkm2 is a glycolytic isoenzyme of the pyruvate kinase. As pyruvate kinase catalyzes the last step within glycolysis, the dephosphorylation of phosphoenolpyruvate to pyruvate is responsible for net ATP production [57]. Once the Pkm2 product pyruvate is produced, it either enters the tricarboxylic acid (TCA) cycle for further production of ATP under aerobic conditions, or is converted to lactic acid or ethanol under anaerobic conditions. Overexpression of Pkm2 modRNA in CMs limits the action of the dominant isoform Pkm1, as both compete for the substrate. High expression of Pkm2 modRNA induces the build-up of upstream glycolysis intermediates, which channel them to the PPP pathway. We showed significant upregulation of G6pd, the first rate-limiting enzyme of PPP after Pkm2 modRNA expression, in the heart and in vitro. We used ^13^C isotopic tracers to investigate metabolic flux by adding ^13^C glucose to the media of neonatal rat CMs [13]. The data showed elevated levels of PPP metabolites and increased ribonucleotide synthesis. The activated PPP reduced the oxidative stress in the mouse heart post-MI and _CMS_Pkm2 modRNA injection. 

It is known that IGF-1 signaling will be activated in the heart post-MI, and IGF-1 expression or upregulation can lead to the formation of epicardial adipose tissue (EAT) post-MI. EAT, a dynamic tissue, plays a critical role in the pathophysiology of the heart. Excessive epicardial fat deposition around the heart may trigger the production of several adipocytokines and chemokines through the activation of various paracrine and autocrine signaling pathways, resulting in the development of atherosclerotic plaques in the coronary vessels [73]. Recently Zangi et al. showed that the application of Cre modRNA in the form of biocompatible gel onto the surface hearts of WT1 flx/flx Rosa26 Tomato mice (lineage tracing experiment) revealed that IGF-1 modRNA stimulated the differentiation of epicardium-derived cells (EPDCs) into adipocytes [32]. Similarly, dominant-negative IGF-1 receptor antagonists modRNA application as a gel directly onto cardiac tissue significantly decreased EAT formation and induced cardiac function post-MI. Using modRNA as a gel to synthesize protein or inhibit protein expression on heart surfaces helps in studying the role of epicardium or epicardial cells in different heart diseases. This tool will be beneficial for developing therapeutics using modRNA application on the epicardium.

Ceramidases hydrolyze ceramide to generate free fatty acids and sphingosine, which is then phosphorylated by sphingosine kinase (Sphk) to generate sphingosine 1-phosphate (S1P), a pro-survival lipid mediator with both intra- and extracellular functions [72]. Mutation in AC leads to ceramidase deficiency and causes a lysosomal storage disease called Farber lipogranulomatosis [65]. AC is required during development and plays a vital role in pathophysiology. A high level of ceramide in the heart post-MI is known to worsen cardiac remodeling; inhibiting ceramide synthesis to reduce the pro-apoptotic effect of ceramide has been recommended [63]. AC modRNA expression reduced ceramide synthesis in the heart and in vitro heart cell culture model [36]. AC modRNA reduced the caspase activity in the cell and, in turn, induced the pro-survival pathways and inhibited cell apoptosis in the heart post-MI.

## 9. modRNA in Cardiac Angiogenesis

In the last few decades, multiple clinical trials with VEGF were conducted to induce revascularization in the ischemic heart and cardiac repair by injecting recombinant protein, naked cDNA, non-viral plasmid, or adenoviral plasmid through intracoronary, intravenous or intramyocardial routes [74,75,76,77,78,79,80]. These studies yielded inconsistent results and produced side effects that might be due to the short half-life of recombinant VEGF in plasma, genomic integration, an immune response against the vector, a limitation of controlled release, and off-target side effects associated with systemic delivery, development of edema or angioma due to AAV-based prolonged expression of VEGF. In the pioneering work by Zangi et al., it has been shown that VEGF-modified mRNA (m1Ψ) can drive multipotent human ES cells or epicardial cells—derived heart progenitors—toward a vasculogenic cell fate both in vitro and in vivo [35]. VEGF-modified mRNA drive cell fate decisions post-MI. Myocardial delivery of a single dose of VEGF modRNA in vivo post-MI leads to vascular regeneration and a cell fate switch of the endogenous epicardial progenitors (quiescent WT1^+^ adult epicardial cells) toward vascular and myocardial cell fates. This substantially adds endothelial cells to provide oxygen and nutrient in an infarcted heart and induces a reparative signal. Using different independent lineage tracing systems, the authors showed that low EPDCs differentiate into CMs; however, with limitations of genetic lineage tracing approaches and lack of CM differentiation in the in vitro clonal assay, further studies are required to support this conclusion. As a result, mice had significantly improved cardiac function, reduced fibrosis, and long-term induced survival [35]. 

The intradermal delivery of VEGF-A modRNA or buffered saline placebo in men with type 2 diabetes mellitus at randomized sites on the forearm caused mild injection-site reactions without any notable side effects. VEGF-A protein levels were induced at mRNA-treated sites versus placebo in 4–24 h post-administration with no clinically significant elevation in plasma VEGF-A levels [81]. This enhanced basal skin blood flow in the patient. This showed VEGF- A modRNA was well tolerated, led to local functional VEGF-A protein expression, and suggests VEGF-A modRNA may have therapeutic potential for regenerative angiogenesis. Using multi-parametric photoacoustic microscopy, researchers showed that injection of VEGF-A modRNA intradermally, induced persistent, marked, and dose-dependent vasodilation, blood flow upregulation, and new blood vessel formation in a mouse model of the diabetic wound [81]. The consecutive injection of VEGF-A modRNA improved vascularization and tissue oxygenation of the wound bed, leading to enhanced re-epithelialization during the early phase of diabetic wound healing in mice [82]. Lately, a population pharmacokinetic and pharmacodynamics-based model was established to quantify the effect of VEGF-A modRNA injections in mice on the dynamics of wound healing with different doses, dosing time points, and the number of doses. Administration of VEGF-A modRNA induced a persistent expedition of wound healing, depending on the accumulated dose, and decreased the time to reach 50% of wound healing by up to 5 days [83].

## 10. Future of modRNA Therapy in Heart Diseases

The outlook of using nucleic acids as therapeutics for cardiac regeneration and heart diseases is very impressive, but also poses a series of novel challenges in biological understanding and clinical application. Cells typically take up mRNA via endocytosis; endosomal membranes containing TLRs recognize single-stranded RNA as foreign and activate an innate immune response. In the case of modRNA, the immune response is reduced significantly, but has not been studied well in terms of clinical set up and safety profile in humans. The second obstacle to the translation of the imported mRNA is its degradation by RNase, while using pseudouridine results in a lower immune response and increased stability to RNase [28,84]. However, still, modRNA degrades very fast when injected intravenously. Further modifications or encapsulation material should be developed to make modRNA stable against RNases and in blood. The third is the delivery of modRNA, where a suitable delivery method is required to transfect cardiac cells in vitro or in the heart to obtain a more significant functional result. A high level of modRNA transfection in vitro can be achieved by mixing modRNA with positively charged polymers, saline, or lipids. RNAiMAX, a positively charged transfection reagent, is popular for delivering RNA molecules in vitro. However, the RNAiMAX reagent is known to induce cell death in the heart, which makes it unsuitable for heart diseases as a gene delivery material [33]. Recently it was reported that the myocardial delivery of modRNA (m1Ψ) in saline–citrate buffer (sucrose–citrate buffer) induced significant cell transfection (both CMs and non-CMs) [33]. The low quantity of naked modRNA is enough to transfect more than 25% of the left ventricle, showing enhanced transfection efficiency of modRNA that will help in use as therapeutics for heart diseases. The major limitation of this delivery method to patients in the clinic is invasiveness; however, developing non-invasive cardiac-targeted delivery platforms will be beneficial and will need time.

The fourth obstacle is cell specificity; AAV is well known as a cell-specific gene delivery platform to express genes into the heart in animal models using cell-specific promoters. However, in the case of modRNA, there was no prior system. Recently we developed a CM-specific modRNA expression tool, the first of its kind. The system works very well in vitro and in vivo in mice models of heart diseases [13]. This work opens the door to delivering functional gene or genes in the form of modRNA exclusively in CMs to induce cardiac regeneration or improve CM function in large animal models or patients. This tailor-made cell-specific expression platform will remove the non-specific effects of gene therapy, which can be detrimental and severely affect the recovery process. This platform will help to develop cell-specific therapeutics for heart diseases and other organs. One of the peculiar characteristics of modRNA is that multiple modRNAs can be used to transfect the cell, and cells take all modRNAs and translate them [13]. This allows us to express multiple genes simultaneously in a more controlled and stable way to obtain improved cardiac function in heart disease. Gene expression using modRNA therapeutics offers new avenues. It removes the complications associated with DNA and protein-based gene therapy, paving the way to applications in HF, MI, and other cardiac diseases that appeared distant until a short time ago. While the potential use of modRNA as a gene therapy tool in the clinic is still early, the transient expression of modRNA might be ideal for cardiac regeneration. However, its transient expression characteristic is a hurdle for continuing protein replacement therapy. If the above limitations of modified mRNA are addressed in the future, then it will find a leading place in the current ranks of a new generation of AAV, small molecules, recombinant proteins, antibodies, and peptide vectors for cardiovascular therapeutics.

## Figures and Tables

**Table 1:** **Vectors used in the cardiovascular system to deliver gene therapies: vector characteristics, application and limitations.**

Sr. No.	Character	Plasmid	Protein	Adenovirus	Lentivirus	AAV	ModRNA
1	Genome type	DNA	Protein (amino acids)	Double stranded DNA	DNA	Single stranded RNA	Single stranded mRNA
2	Size (diameter)	----	----	100	90 nm	20 nm	----
3	Expression duration	1–4 weeks	Hours	1–4 weeks	Long-term	Long-term (months to years)	7–12 days
4	Peak expression in the heart	2–4 days	Hours	2–4 days	4–6 days	2–4 weeks	1–2 days
5	Transfection	Low	----	High	Low	High	High
6	Synthesis time	Short–moderate	Short–moderate	Moderate	Moderate	Moderate	Short
7	Immune response	Mild	No	Severe	Moderate	Mild	Low
8	Gene insert	Limited	----	1–2 genes	2–8 genes	1–2 genes	Unlimited
9	Genomic integration	Yes	No	Yes	Yes	Yes	No
10	Tissue or cell specificity	No	No	No	No	Yes	Yes/No
11	Delivery	Lipid	Direct	Direct	Lipid	Direct	Lipid, saline, nanomaterial
12	Delivery method	Direct	Direct or IV	Direct	Direct	Direct or IV	Direct
13	Clinical application	Very low	Moderate	Very low	Very low	Moderate to high	High
14	Human clinical trials in heart diseases (NCT02935712)	----	----	----	----	<5	1

**Table 2:** **Published work showing use of modRNA based gene delivery for heart diseases and regeneration.**

Sr. No.	Publication	Gene/Genes	Study and Role	Animal/Cells
1	Chen et al. [39]	aYAP	Reduction of cardiac inflammation and hypertrophy	Cardiac cells/Mice
2	Sultana et al. [37]	Luc/GFP	Optimization of 5’ untranslated region and modRNA expression in the heart	Cardiac cells/Mice
3	Magadum et al. [13]	Pkm2	Cardiomyocyte-specific modRNA expression, cardiomyocyte proliferation and cardiac regeneration	Cardiac cells/Mice
4	Carlsson et al. [40]	VEGFa	Increase of angiogenesis, reduction of fibrosis and improvement of cardiac function post MI	Cells, Pig, Monkey
5	Hadas et al. [36]	Acid ceramidase	Inhibition of cardiomyocyte apoptosis, improved cardiac function	Cardiac cells/Mice
6	Hadas et al. [41]	Luc/GFP	Optimization of modified mRNA in vitro synthesis protocol for heart gene therapy	Cardiac cells/Mice
7	Singh et al. [42]	EGFP, mCherry, Fluc	Microencapsulated modRNA expression in the heart	Mice, Pig
8	Magadum et al. [12]	Mutated FSTL1	Cardiomyocyte proliferation, reduction of scar size and improvement of cardiac function post-MI	Cardiac cells/Mice
9	Sultana et al. [33]	Luc/GFP	Optimization of modRNA expression	Cardiac cells/Mice
10	Turnbull et al. [15]	EGFP	modRNA expression in the heart	Rat and Pig
11	Zangi et al. [32]	IGFR, DN-IGF-1R	Inhibition of adipogenic differentiation post-MI	Mice
12	Kondrat et al. [43]	Luc/GFP	modRNA synthesis	In vitro/Mice
13	Turnbull et al. [44]	EGFP	Lipidoid mRNA nanoparticles protocol	Rodents
14	Huang et al. [34]	IGF-1	Inhibition of cardiomyocyte apoptosis and survival cardiomyocytes	Mice
15	Lui at al. [45]	VEGFa	Promotion of Isl1^+^ to endothelial cell fate, survival and proliferation of Isl1^+^ progenitors	Mice
16	Zangi et al. [35]	VEGFa	Inducement of vascular regeneration and cellular fate switch	Cardiac cells/Mice

## Data Availability

Not applicable.

## Abbreviations

MI	myocardial infarction
HF	heart failure
iPSCs	induced pluripotent stem cells
hiPSC-CMs	human-induced pluripotent stem cell-derived cardiomyocytes
ROS	reactive oxygen species
miRNA	microRNA
CVD	cardiovascular diseases
CM	cardiomyocyte
FDA	Food and Drug Administration
ARCA	anti-reverse cap analog
PPP	pentose phosphate pathway
IGF-1	insulin growth factor 1
YAP	yes-associated protein
TNF-α	tumor necrosis factor-α
Pkm2	pyruvate kinase isozyme 2
G6PD	glucose-6-phosphate dehydrogenase
ihCD25	inactive human CD25
